# Structural Phase Transition of ThC Under High Pressure

**DOI:** 10.1038/s41598-017-00226-4

**Published:** 2017-03-07

**Authors:** Cun Yu, Jun Lin, Ping Huai, Yongliang Guo, Xuezhi Ke, Xiaohe Yu, Ke Yang, Nana Li, Wenge Yang, Baoxing Sun, Ruobing Xie, Hongjie Xu

**Affiliations:** 1 Shanghai Institute of Applied Physics, Chinese Academy of Sciences (CAS), Shanghai, 201800 China; 2 Department of Physics, East China Normal University, Shanghai, 200241 China; 3 Shanghai Synchrotron Radiation Facility, Shanghai Institute of Applied Physics, Chinese Academy of Sciences (CAS), Shanghai, 201204 China; 4 Center for High Pressure Science and Technology Advanced Research (HPSTAR), Shanghai, 201203 P. R. China; 5 High Pressure Synergetic Consortium (HPSynC), Geophysical Laboratory, Carnegie Institution of Washington, Argonne, Illinois 60439 USA

## Abstract

Thorium monocarbide (ThC) as a potential fuel for next generation nuclear reactor has been subjected to its structural stability investigation under high pressure, and so far no one reported the observation of structure phase transition induced by pressure. Here, utilizing the synchrotron X-ray diffraction technique, we for the first time, experimentally revealed the phase transition of ThC from B1 to P4/nmm at pressure of ~58 GPa at ambient temperature. A volume collapse of 10.2% was estimated during the phase transition. A modulus of 147 GPa for ThC at ambient pressure was obtained and the stoichiometry was attributed to the discrepancy of this value to the previous reports.

## Introduction

As the development of the advanced nuclear reactor, thorium energy has brought wide attentions. Thorium is more abundant than uranium in the crust of the Earth. Natural thorium can be formed into U-233, a well-known nuclear fuel material after absorbing a neutron, which makes it a very promising fuel for a breeder reactor^1^. Among the common compounds of thorium, thorium monocarbide (ThC) has a good thermal stability (melting point: ~2625 K) and thermal conductivity (29 W/mK) as a candidate reactor fuel.

For the concern of its applications, the high pressure stability of ThC has stimulated many interests of theorists and experimentalists. At ambient conditions, thorium monocarbide (ThC) forms a NaCl type structure (denoted as B1 phase). The high pressure study of ThC has been carried out since 1980s. Gerward *et al.* ^2, 3^ has investigated the structural stability up to 50 GPa, and did not observe any structural transition over the whole pressure range. It was not until quite recently, theoretical predictions based on first-principle calculations reported that the ambient B1 phase could be transformed to other phases at different^4, 5^ pressures. Sahoo *et al.* ^4^ reported a pressure phase transition sequence of B1 → Pnma → Cmcm → CsCl type (B2) at hydrostatic pressures of ~19 GPa, 36 GPa, and 200 GPa, respectively. However, Guo *et al*.^5^ proposed that the B1 phase can be transformed to P4/nmm and then B2 phases at 60 GPa and 120 GPa respectively. Therefore, the question still remains unanswered: “Dose a phase transition exist in ThC under high pressure? Which structural phase transition route should it follow?” In our study, we confirmed the phase transition (B1-P4/nmm) in ThC at the pressure near 58 GPa by using a high pressure diamond anvil cell combined with the micro-focused X-ray diffraction technique, accompanying with a large (10.2%) volume collapse at the transition pressure.

## Results and Discussion

**Figure 1:**
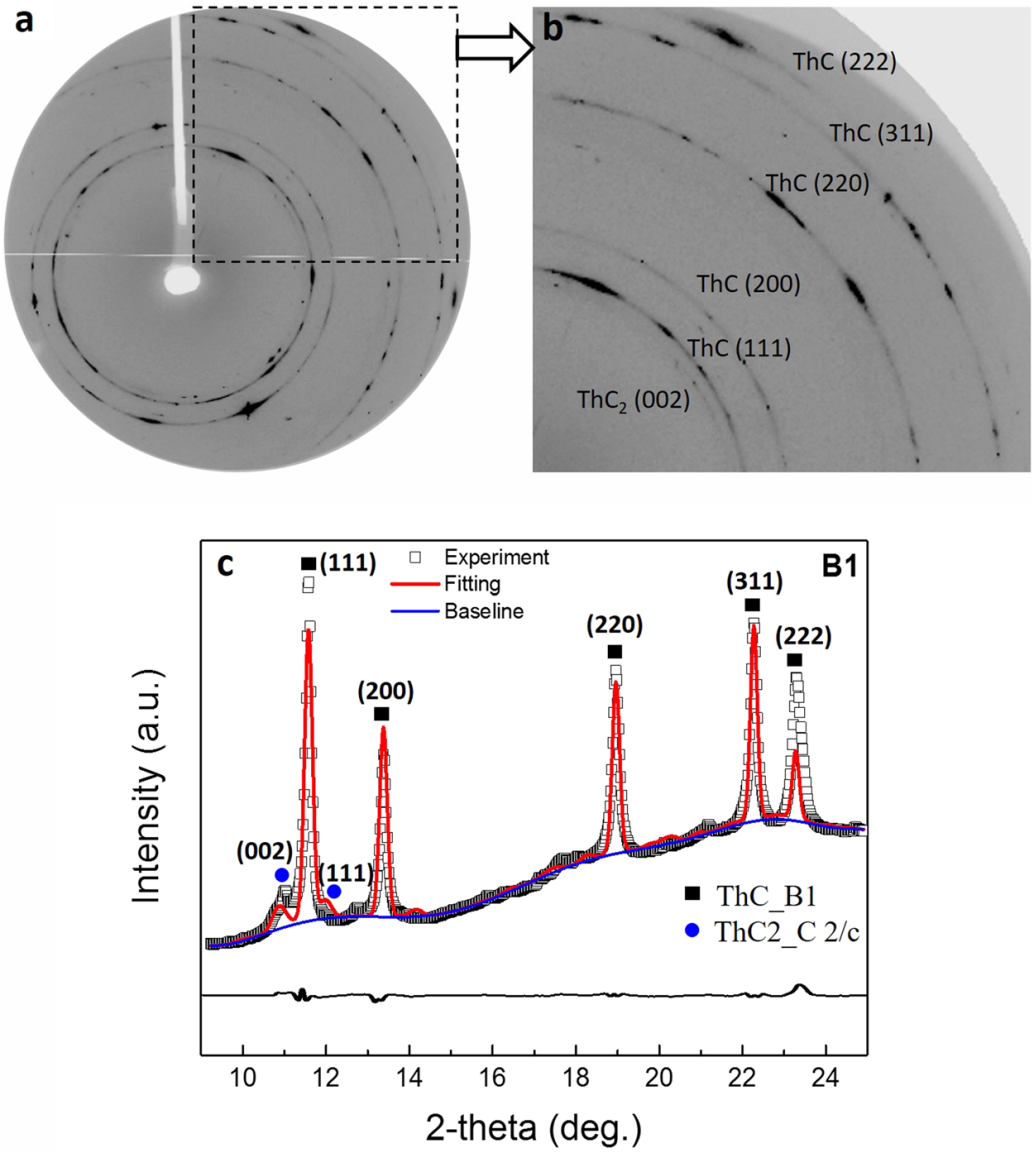
X-ray diffraction (XRD) result of ThC powder at initial high pressure 1.23 GPa. (**a**) The original 2D diffraction pattern. The indexes are shown on (**b**). The enlarged area from (**a**) with indexation of diffraction peaks. (**c**) The corresponding integrated 1D diffraction pattern and Rietveld refinement result (red line).

After the sample was loaded, there was an initial pressure of 1.2 GPa in the DAC. A typical two-dimensional (2D) diffraction pattern is shown in Fig. 1a at the first pressure point 1.2 GPa in the diamond anvil cell. Figure 1b shows the enlarged image of Fig. 1a. The integrated XRD profile is shown in Fig. 1c with the result of Rietveld refinement. This structure coincides with the B1 ThC (NaCl-type, space group Fm-3 m) at room temperature and atmospheric pressure, with d space slightly decreased due to the initial pressure. Besides, a weak peak near (111)_ThC_ was identified as (002) from ThC_2_, a by-product owing to the excess carbon consumed during the synthesis process^6^. The content of ThC_2_ was estimated to be 2–5% by weight.

**Figure 2:**
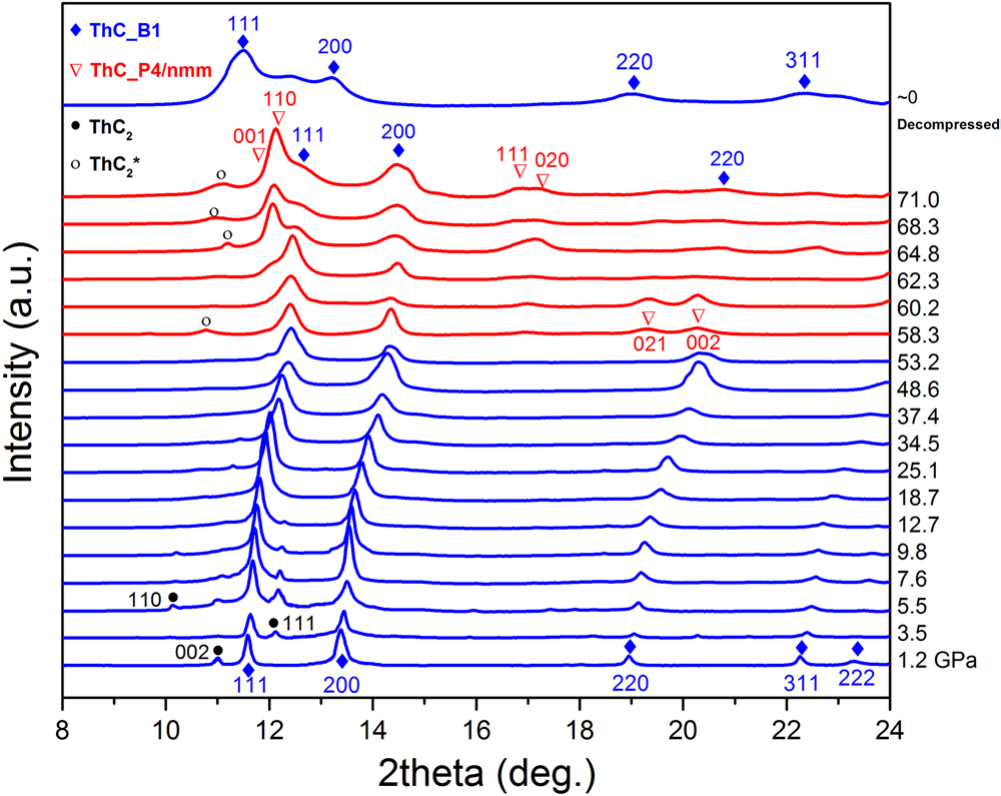
Integrated 1D XRD profiles of ThC powder at various pressures ranging from 1.2 GPa to 71.0 GPa.(Notes: blue diamond represents ThC-B1 phase, red triangle represents ThC-P4/nmm phase, solid black circle represents ThC_2_-C2/c phase and hollow black circle represents ThC_2_-C2/m phase).

Pressure was gradually increased up to 71.0 GPa and then released. The diffraction profiles of ThC at pressures ranged from 1.2–71.0 GPa and back to ambient pressure are shown in Fig. 2 after the background was subtracted. The signals remains the same up to 53.2 GPa except for the major diffraction peaks becoming broader and shifting to larger angles systematically as pressure goes up, indicating the lattices are under distortion, partially resulting from the possible shear stresses in the sample due to the imperfect hydrostaticity of silicon oil. Starting from 58.3 GPa, new diffraction peaks show up at around 17 and 19 degrees, indicating the onset of a new phase. These peaks are identified as (111), (020) and (021) of the P4/nmm ThC. Another evidence of the new phase can be seen at diffraction curves at top 3 pressure points, where on each curve a strong peak appears at a lower angle next to the (111) peak of B1 ThC. One can even trace the origin of this peak back to 53.2 GPa. It is identified as (110) of the P4/nmm phase. The Rietveld refinement of high pressure (71 GPa) diffraction pattern is shown in Fig. S1. Even though from our data we can see the coexistence of the B1 and P4/nmm phase of ThC between 53.2 and 71.0 GPa, which are attributed to the pressure gradient in the sample chamber, the new phase of P4/nmm is undoubtedly discovered. Hence we conclude that ThC has a phase transition at around 58 GPa from B1 to P4/nmm. This is the first time ever that the high pressure transition of ThC has been experimentally reported. Our results agree well with the prediction by Guo *et al*.^5^. After the pressure was released, the sample returned back to the ambient phase B1, as the top curve shows in Fig. 2. Compared to the 1.2 GPa B1 phase, its peaks shift to the smaller angles as d space slightly increases under ambient condition, and become much broader as a result of many crystalline defects produced during the high pressure compression.

The phase transition of ThC under high pressure was previously studied through a first principle calculation^4^, and they found a high pressure structural transition sequence of NaCl type (B1) → Pnma → Cmcm → CsCl type (B2) at hydrostatic pressures of ~19 GPa, 36 GPa, and 200 Gpa, respectively. We checked our experimental data from 1.2 GPa to 53.2 Gpa and found no new peaks. Then we compared the experimental results obtained at higher pressures (>58.3 GPa) with the calculated phases including P4/nmm^5^, Pnma and Cmcm^4^, respectively, it was found that only P4/nmm phase agreed well with our experimental results (refer to Fig. S1 and R1). To compare the difference of these three phases, we calculated the atomic densities of P4/nmm, Pnma and Cmcm, which are 70.2%, 35.3% and 25.8% respectively. Among them, P4/nmm is the densest and would remain the best structural integrity under high pressure.

As mentioned previously, our ThC sample was mixed with small amount of ThC_2_, whose characteristic signals are weak yet visible. Its several peaks appearing on the lower pressure curves were identified to originate from the ambient monoclinic phase (space group C2/c), based on another work of our high pressure experiments on pure ThC_2_^7^. The high pressure phase of ThC_2_ can be seen at the top few pressure points, as marked in Fig. S1. It is worthy to point out that this new high pressure structure of ThC_2_ has never been reported before, experimentally or theoretically as to our best knowledge. The details of the structural information of ThC_2_ would be discussed in another article^7^.

**Figure 3:**
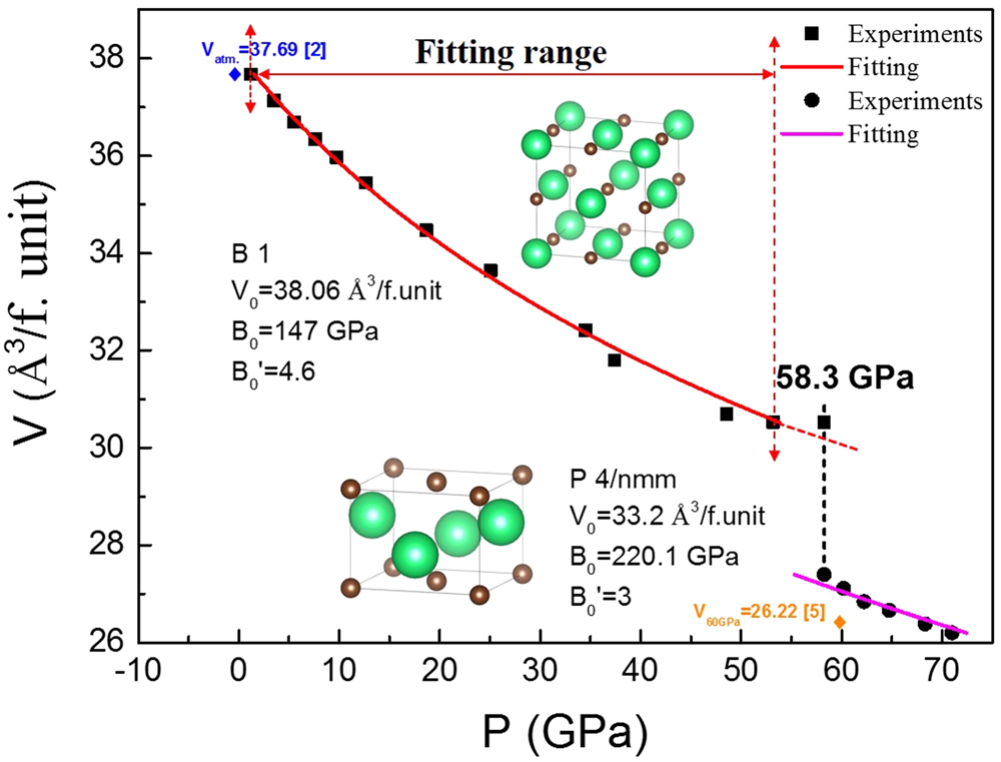
The pressure-volume data of ThC-B1 and P4/nmm phases up to 71.0 GPa. The solid lines represent the fitted result using 3rd order Birch-Murnaghan equation of state. The two diamond symbols represent the values from two previous reports respectively for comparison.

The unit cell volumes of ThC were calculated based on the Rietveld refinement results at entire pressure range as shown in Fig. 3. The abrupt drop of volume at ~58 GPa clearly separates the data into two groups, undoubtedly suggesting the occurrence of a first order phase transition. The data can be fitted with the third-order Birch-Murnaghan equation of state (EOS)^8^ for B1 (before transition) and P4/nmm (after transition) phase separately:

1 $$p=1.5{B}_{0}[{(\frac{v}{{v}_{0}})}^{\frac{-7}{3}}-{(\frac{v}{{v}_{0}})}^{\frac{-5}{3}}]\{1-0.75(4-{{B}_{0}}^{\text{'}})[{(\frac{v}{{v}_{0}})}^{\frac{-2}{3}}-1]\}$$

where *v*/*v*_0_ is the ratio of unit cell volume at pressure p to that at ambient pressure. B_0_ is the bulk modulus at ambient condition, and B_0_′ is its pressure derivative. The least-square fittings yield B_0_ = (147 ± 3) GPa, and B_0_′ = (4.6 ± 0.1) for B1 phase between 1.2 and 53.2 GPa, and B_0_ = (220 ± 4) GPa, and B_0_′ = (3.0 ± 0.1) for P4/nmm phase between 58.3 and 71.0 GPa. The percentage of unit cell volume collapse from B1 to P4/nmm at 58.3 GPa is ~10.2%.

In Table 1, we summarize and compare the lattice parameters and compressibility of ThC from present study and previous experimental and theoretical results^2–5, 9–13^. It is worthy to note that the bulk modulus (147 GPa) of B1 phase in our study is much higher than the reported value (109 GPa^2, 3^). The discrepancy is possibly due to the difference of stoichiometry as suggested by J. Staun Olsen *et al*.^14^. In Gerward’s study^2^, they estimated that their sample should have a composition corresponding to ThC_0.76_. Based on the work of Pialoux and Zaug^15^, the stoichiometry of our sample is estimated to be Th:C = 1:0.95, different from Gerward’s. Furthermore, J. Staun Olsen *et al*.^14^ summarized the results for the uranium and thorium compounds respectively and found that smaller lattice parameters generally lead to larger bulk moduli with same crystal structure. The lattice parameters of thorium nitrides (ThN) and thorium sulphides (ThS) are 5.1666 Å and 5.6851 Å, respectively. In the present study, this value for ThC is 5.3397 Å which lies between that of ThN and ThS. As the bulk moduli of ThN^16^ and ThS^17^ are reported to be 175 GPa and 145 GPa respectively, it is reasonable to conjecture that the bulk modulus of ThC would have the value that is smaller than 175 but larger than 145 GPa, exactly where our result (147 GPa) fits. (A full list of the obtained structural parameters for both the B1 and the high-pressure ThC (P4/nmm) phases is shown in Table S1). Another minor factor that might contribute to the discrepancy is the different degree of the hydrostaticity of the pressure medium.

**Table 1:** Lattice parameters and compressibility of ThC at ambient and high pressure obtained from experiments and calculations.

Space group	Dimension & Compressibility	Present work (300 K)	Other works	
			Experimental	Theoretical
B1	a (Å)	5.3397	5.3218^2^ 5.3208^3^	5.3480^4^ 5.352^5^
	V_0_ (Å^3^/f. unit)	38.06	37.68^2^ 37.66^3^	38.24^4^ 38.32^5^ 39.10^8^ 38.06^9^ 38.08^10^ 37.96^11^ 38.75^12^
	B_0_ (GPa)	147	109^#,2,3^	137^4^ 132^8,9^ 134^10^ 135^11^ 121^12^
	B_0_′	4.6	3.1^2^	3.09^4^ 2.95^4^ 2.88^9^ 3.00^11^ 3.31^12^
P 4/nmm	a (Å)	4.205*	—	4.509^5^ 4.134*
	c (Å)	3.099*	—	3.683^5^ 3.060*
	V_0_ (Å^3^/f. unit)	33.2	—	37.45^5^
	B_0_ (GPa)	220.1	—	229.6^5^
	B_0_′	3	—	—

All calculation results were obtained at zero pressure and 0 K. *Obtained at 58.3 GPa. ^#^The stoichiometry is ThC_0.76_.

In Table 1, we compare the major parameters of the dimensions and compressibility of ThC between our experimental results with previous reports. At ambient pressure, the lattice parameter of B1 phase measured in our study is slightly larger than the other experimental values which could be attributed to the compositions of the sample. In ref. 2, the stoichiometry of ThC was estimated to be Th:C = 1:0.76. In our study, we estimate that this ratio to be ~1:0.95. The excess carbon will lead to a larger lattice parameter. At elevated pressure where P4/nmm phase exists, there are two groups of values provided by Guo *et al*. One is obtained at zero pressure^5^, while the other is calculated at 58.3 GPa (marked as *), same as the experimental condition. The lattice parameter calculated at zero pressure are noticeably larger than the experimental values obtained at high pressure, which is reasonable. The theoretical values calculated at same pressure as the experimental values agree quite well with them within 2% difference. The discrepancy on unit cell volume between the experimental and theoretical values is about 13%, and around 4% on the bulk modulus.

## Conclusion

The crystal structure of ThC under high pressure was studied up to 71.0 GPa with a diamond anvil cell and micro-focused X-ray beam. Diffraction patterns revealed a first order phase transition from B1 to P4/nmm in ThC at ~58 GPa as the pressure increased. The result validates the prediction based on the first principles calculation by Guo *et al*. The bulk modulus of B1 phase is found to be ~147 GPa and the volume collapse during phase transition was estimated to be 10.2%.

## Methods

Thorium monocarbide (ThC) was synthesized using thorium dioxide powder and natural graphite powder as starting materials by carbon thermal reduction method (SDCTM). The ThO_2_ and graphite powders were mixed with C/ThO_2_ molar ratio of 3.0 and followed with ball-milling for 2 h in ethanol. After that, the slurry was dried at 100 °C for 48 h in a vacuum drier. Then the dried mixture was pressed into pellets with 5 mm in diameter and 10 mm in height. Finally, the green pellets were sintered at 1950 °C with the vacuum of 1.3 × 10^−3^ Pa for 30 min. After cooling down, the sintered specimens were immersed in cyclohexane to prevent oxidizing and hydrolyzing.

In order to obtain fine powder for X-ray diffraction (XRD) characterization, the bulk sample was grinded into sub-micro sized particles (200–300 nm) within silicon oil to avoid deliquesce.

A Mao-Bell type symmetric diamond-anvil cell with a pair of 200 μm culets was used to generate high pressure environment for thorium monocarbide. A hole of 80 μm in diameter and 30 μm in thickness was drilled at the center of the pre-indented stainless steel gasket as the sample chamber. A small piece of sample was loaded in the center of the chamber with three small ruby spheres (3–5 μm in diameter) at different locations in the chamber for monitoring the pressure distribution inside the sample chamber. Silicon oil was used as pressure transmitting medium.

*In-situ* high-pressure XRD measurements were carried out at BL15U station at Shanghai Synchrotron Radiation Facility (SSRF). The monochromatic x-ray beam with wavelength 0.6199 Å was focused to a rectangle of ~3 μm (vertical) × 2.5 μm (horizontal) measured by full width at half maximum (FWHM). The diffraction patterns were collected using a MarCCD 165 image plate, with typical exposure time of 20 to 60 seconds. Each diffraction pattern was collected after the pressure was adjusted and stabilized to ensure steady pressure during XRD measurements. The two-dimensional diffraction patterns were integrated into one dimensional profiles of intensity versus 2-theta with FIT2D program^18^, followed by the GSAS structural Rietveld refinement^19^.

## Electronic supplementary material

Supplementary information
