# Supplementary material for: Structural Phase Transition of ThC Under High Pressure
Source: Sci Rep. 2017 Mar 7;7:96. doi: 10.1038/s41598-017-00226-4 (PMC5428025; doi:10.1038/s41598-017-00226-4)
Supplement: Supplementary file 1 — Supplementary information [file 41598_2017_226_MOESM1_ESM.pdf]

Supplementary Materials

Structural Phase Transition of ThC Under High Pressure

Cun Yu[1], Jun Lin[1], Ping Huai[1], Yongliang Guo[1,2], Xuezhi Ke[2], Xiaohe Yu[1], Ke Yang[3], Nana Li[4], Wenge Yang[4, 5], Baoxing Sun[1], Ruobing Xie[1,*], Hongjie Xu[1]

[1]*Shanghai Institute of Applied Physics, Chinese Academy of Sciences (CAS), Shanghai, 201800, China;*

[2]*Department of Physics, East China Normal University, Shanghai 200241, China;*

[3]*Shanghai Synchrotron Radiation Facility, Shanghai Institute of Applied Physics, Chinese Academy of Sciences (CAS),Shanghai 201204, China;*

[4]*Center for High Pressure Science and Technology Advanced Research (HPSTAR), Shanghai 201203, P. R. China;*

[5]*High Pressure Synergetic Consortium (HPSynC), Geophysical Laboratory, Carnegie Institution of Washington, Argonne, Illinois 60439, USA*

*To whom correspondence should be addressed. E-mail: xieruobing@sinap.ac.cn

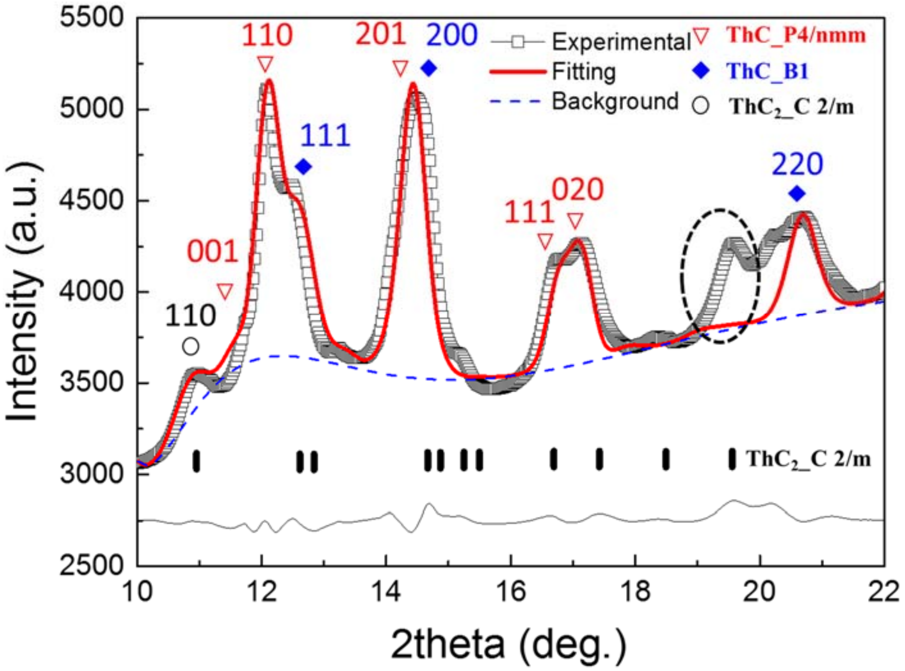

Fig. S1 Rietveld refinement of diffraction file under high pressure (71 GPa)

It should be noted that those black short markers under the diffraction pattern represent the Bragg peaks' positions of high pressure ThC_2 (space group: C2/m). The unfitted peak as circled by dotted line may be from the reflection (200) of gasket (stainless steel).

Table S1. Lattice parameters of both B1 and P4/nmm phases under different pressures

Pressure (GPa)	ThC-B1	ThC-P4/nmm	
	a	a	c
**1.2**	5.321(4)		
**3.5**	5.295(8)		
**5.5**	5.274(8)		
**7.6**	5.257(8)		
**9.8**	5.240(1)		
**12.7**	5.214(4)		
**18.7**	5.165(9)		
**25.1**	5.124(4)		
**34.5**	5.061(4)		
**37.4**	5.029(2)		
**48.6**	4.970(5)		
**53.2**	4.961(4)		
**58.3**	4.961(4)	4.204(9)	3.099(3)
**60.2**	4.960(8)	4.188(5)	3.091(1)
**62.3**	4.937(1)	4.158(7)	3.081(2)
**64.8**	4.940(8)	4.157(8)	3.061(2)
**68.3**	4.929(4)	4.150(1)	3.040(5)
**71.0**		4.163(8)	3.000(2)
